# Supplementary material for: Duration of intervals in the care seeking pathway for lung cancer in Bangladesh: A journey from symptoms triggering consultation to receipt of treatment
Source: PLoS One. 2021 Sep 10;16(9):e0257301. doi: 10.1371/journal.pone.0257301 (PMC8432814; doi:10.1371/journal.pone.0257301)

PLOS ONE

Duration of intervals in the care seeking pathway for lung cancer in Bangladesh: a journey from symptoms triggering consultation to receipt of treatment

--Manuscript Draft--

**Manuscript Number:**	PONE-D-20-39263
**Article Type:**	Research Article
**Full Title:**	Duration of intervals in the care seeking pathway for lung cancer in Bangladesh: a journey from symptoms triggering consultation to receipt of treatment
**Short Title:**	Duration of intervals in the care seeking pathway for lung cancer in Bangladesh
**Corresponding Author:**	Adnan Ansar, MBBS, MPH, PhD (Candidate)<br>La Trobe University<br>Melbourne, VIC AUSTRALIA
**Keywords:**	lung cancer; Bangladesh; care seeking pathway; triggering symptom; duration of interval.
**Abstract:**	Introduction:<br>Timeliness in seeking care is critical for lung cancer patients' survival and better prognosis. This study investigated the symptoms triggering healthcare seeking, preferred healthcare providers, and the duration of intervals in the lung cancer care pathway of patients in Bangladesh.<br>Methods:<br>A cross-sectional study was conducted in three tertiary care hospitals in Bangladesh among diagnosed lung cancer patients through face-to-face interview and medical record review. Time intervals from onset of symptom and care seeking events were estimated and compared between those who sought initial care from different providers using Wilcoxon rank sum tests.<br>Results:<br>Among 418 study participants, the majority (90%) of whom were males, with a mean age of 57 ±9.86 years, cough and chest pain were the most common (23%) combination of symptoms triggering healthcare seeking. About two-thirds of the total respondents (60%) went to informal healthcare providers as their first point of contact. Living in rural areas, lower levels of education and lower income were associated with seeking care from such providers. The median duration between onset of symptom to confirmation of diagnosis was 121 days, between confirmation of diagnosis and initiation of treatment was 22 days, and between onset of symptom and initiation of treatment was 151 days. Pre-diagnosis durations were longer for those who had sought initial care from an informal provider ($p<0.05$).<br>Conclusion:<br>Time to first contact with a health provider was shorter in this study compared to other countries but utilizing informal healthcare providers caused delays in diagnosis and initiation of treatment. Creating awareness of lung cancer symptoms and encouraging people to seek care from a formal healthcare provider may reduce the overall duration of the care seeking pathway.
**Order of Authors:**	Adnan Ansar, MBBS, MPH, PhD (Candidate)
	Virginia Lewis
	Christine Faye McDonald
	Chaojie Liu
	Muhammad Aziz Rahman
**Additional Information:**	
**Question**	**Response**
**Financial Disclosure**<br><br>Enter a financial disclosure statement that	The authors received no specific funding for this work.

Powered by Editorial Manager® and ProduXion Manager® from Aries Systems Corporation

describes the sources of funding for the work included in this submission. Review the submission guidelines for detailed requirements. View published research articles from *PLOS ONE* for specific examples.<br><br>This statement is required for submission and **will appear in the published article** if the submission is accepted. Please make sure it is accurate.<br><br>**Unfunded studies**<br>Enter: *The author(s) received no specific funding for this work.*<br><br>**Funded studies**<br>Enter a statement with the following details:<br>• Initials of the authors who received each award<br>• Grant numbers awarded to each author<br>• The full name of each funder<br>• URL of each funder website<br>• Did the sponsors or funders play any role in the study design, data collection and analysis, decision to publish, or preparation of the manuscript?<br>• **NO** - Include this sentence at the end of your statement: *The funders had no role in study design, data collection and analysis, decision to publish, or preparation of the manuscript.*<br>• **YES** - Specify the role(s) played.<br><br>* typeset	
**Competing Interests**<br><br>Use the instructions below to enter a competing interest statement for this submission. On behalf of all authors, disclose any competing interests that could be perceived to bias this work—acknowledging all financial support and any other relevant financial or non-financial competing interests.<br><br>This statement **will appear in the published article** if the submission is accepted. Please make sure it is accurate. View published research articles from *PLOS ONE* for specific examples.	The authors have declared that no competing interest exist.

**NO authors have competing interests**<br><br>Enter: *The authors have declared that no competing interests exist.*<br><br>**Authors with competing interests**<br><br>Enter competing interest details beginning with this statement:<br><br>*I have read the journal's policy and the authors of this manuscript have the following competing interests: [insert competing interests here]*<br><br>* typeset	
**Ethics Statement**<br><br>Enter an ethics statement for this submission. This statement is required if the study involved:<br><br>• Human participants<br>• Human specimens or tissue<br>• Vertebrate animals or cephalopods<br>• Vertebrate embryos or tissues<br>• Field research<br><br>Write "N/A" if the submission does not require an ethics statement.<br><br>General guidance is provided below. Consult the submission guidelines for detailed instructions. **Make sure that all information entered here is included in the Methods section of the manuscript.**	This study obtained ethical approval from the University Human Ethics Committee of La Trobe University, Melbourne, Australia (HEC19154); National Institute of Cancer Research & Hospital, Dhaka, Bangladesh (NICRH/Ethics/2019/504); and Ahsania Mission Cancer and General Hospital, Dhaka Bangladesh (DAM/AMCGH/1900-2019-2063). Written informed consent was obtained from participants prior to data collection.

**Format for specific study types**<br><br>**Human Subject Research (involving human participants and/or tissue)**<br>• Give the name of the institutional review board or ethics committee that approved the study<br>• Include the approval number and/or a statement indicating approval of this research<br>• Indicate the form of consent obtained (written/oral) or the reason that consent was not obtained (e.g. the data were analyzed anonymously)<br><br>**Animal Research (involving vertebrate animals, embryos or tissues)**<br>• Provide the name of the Institutional Animal Care and Use Committee (IACUC) or other relevant ethics board that reviewed the study protocol, and indicate whether they approved this research or granted a formal waiver of ethical approval<br>• Include an approval number if one was obtained<br>• If the study involved *non-human primates*, add *additional details* about animal welfare and steps taken to ameliorate suffering<br>• If anesthesia, euthanasia, or any kind of animal sacrifice is part of the study, include briefly which substances and/or methods were applied<br><br>**Field Research**<br><br>Include the following details if this study involves the collection of plant, animal, or other materials from a natural setting:<br>• Field permit number<br>• Name of the institution or relevant body that granted permission	
**Data Availability**<br><br>Authors are required to make all data underlying the findings described fully available, without restriction, and from the time of publication. PLOS allows rare exceptions to address legal and ethical concerns. See the PLOS Data Policy and FAQ for detailed information.	No - some restrictions will apply

Powered by Editorial Manager® and ProduXion Manager® from Aries Systems Corporation

A Data Availability Statement describing where the data can be found is required at submission. Your answers to this question constitute the Data Availability Statement and **will be published in the article**, if accepted.<br><br>**Important:** Stating 'data available on request from the author' is not sufficient. If your data are only available upon request, select 'No' for the first question and explain your exceptional situation in the text box.<br><br>Do the authors confirm that all data underlying the findings described in their manuscript are fully available without restriction?	
**Describe where the data may be found in full sentences. If you are copying our sample text, replace any instances of XXX with the appropriate details.**<br><br>• If the data are **held or will be held in a public repository**, include URLs, accession numbers or DOIs. If this information will only be available after acceptance, indicate this by ticking the box below. For example: *All XXX files are available from the XXX database (accession number(s) XXX, XXX.*).<br>• If the data are all contained **within the manuscript and/or Supporting Information files**, enter the following: *All relevant data are within the manuscript and its Supporting Information files.*<br>• If neither of these applies but you are able to provide **details of access elsewhere**, with or without limitations, please do so. For example:<br><br>*Data cannot be shared publicly because of [XXX]. Data are available from the XXX Institutional Data Access / Ethics Committee (contact via XXX) for researchers who meet the criteria for access to confidential data.*<br><br>*The data underlying the results presented in the study are available from (include the name of the third party*	Data cannot be shared publicly because of ethics committee restriction on this PhD research data. Data are available from University Human Ethics Committee of La Trobe University, Melbourne, Australia contact via Professor Virginia Lewis who meet the criteria for access to confidential data.

Powered by Editorial Manager® and ProduXion Manager® from Aries Systems Corporation

and contact information or URL).<br>• This text is appropriate if the data are owned by a third party and authors do not have permission to share the data.<br><br>* typeset	
Additional data availability information:	

Manuscript
Click here to access/download;Manuscript;Main Manuscript Resubmit PLOS ONE 21.12.2020.docx

Title

Duration of intervals in the care seeking pathway for lung cancer in Bangladesh: a journey from symptoms triggering consultation to receipt of treatment

Author information

Adnan Ansar[1,4]*, Virginia Lewis[1, 2], Christine Faye McDonald[3,4,5], Chaojie Liu[6], Muhammad Aziz Rahman[2,7]

[1] School of Nursing and Midwifery, College of Science Health and Engineering, La Trobe University, Melbourne, Australia

[2] Australian Institute for Primary Care and Aging, La Trobe University, Melbourne, Australia

[3] Department of Respiratory & Sleep Medicine, Austin Health, Melbourne, Australia

[4] Institute for Breathing and Sleep, Melbourne, Australia

[5] University of Melbourne, Melbourne, Australia

[6] School of Psychology and Public Health, La Trobe University, Melbourne, Australia

[7] School of Health, Federation University Australia, Berwick, Australia

*Corresponding author: Adnan Ansar

Email: dr.adnan.ansar@gmail.com; ansar.a@students.latrobe.edu.au

Author Contributions

AA conceived the study and undertook data collection, data analysis and data interpretation with input and supervision from VL, CL, CMcD and MAR. AA prepared the first draft of the manuscript.

All authors provided critical comments and input to revisions to the paper and approved the final manuscript for submission.

ABSTRACT

Background:

Timeliness in seeking care is critical for lung cancer patients' survival and better prognosis. This study investigated the symptoms triggering healthcare seeking, preferred healthcare providers, and the duration of intervals in the lung cancer care pathway of patients in Bangladesh.

Methods:

A cross-sectional study was conducted in three tertiary care hospitals in Bangladesh among diagnosed lung cancer patients through face-to-face interview and medical record review. Time intervals from onset of symptom and care seeking events were estimated and compared between those who sought initial care from different providers using Wilcoxon rank sum tests.

Results:

Among 418 study participants, the majority (90%) of whom were males, with a mean age of 57 ±9.86 years, cough and chest pain were the most common (23%) combination of symptoms triggering healthcare seeking. About two-thirds of the total respondents (60%) went to informal healthcare providers as their first point of contact. Living in rural areas, lower levels of education and lower income were associated with seeking care from such providers. The median duration between onset of symptom to confirmation of diagnosis was 121 days, between confirmation of diagnosis and initiation of treatment was 22 days, and between onset of symptom and initiation of treatment was 151 days. Pre-diagnosis durations were longer for those who had sought initial care from an informal provider ($p<0.05$).

Conclusion:

Time to first contact with a health provider was shorter in this study compared to other countries but utilizing informal healthcare providers caused delays in diagnosis and initiation of treatment. Creating awareness of lung cancer symptoms and encouraging people to seek care from a formal healthcare provider may reduce the overall duration of the care seeking pathway.

INTRODUCTION

Lung cancer is an aggressive disease with poor survival. As the leading cause of cancer death, it has a higher incidence and mortality rate than any other cancer globally [1]. According to the World Cancer Report 2020, there were 2.1 million new cases and 1.8 million deaths in 2018 due to lung cancer. Early stage diagnosis of lung cancer may allow surgical resection, the most effective treatment, with the five-year survival rate being as high as 50-70% [2-4] if diagnosed at Stage I. However, around 75% of people present at an advanced stage of their disease [5], where the overall survival is only 10-20% [1] and 1-5% if diagnosed at Stage IV [2-4].

Timeliness is an important dimension of health care quality. The importance of timely diagnosis and treatment of lung cancer, and the need for indicators to measure timeliness of care are increasingly discussed in the literature. Guidelines for lung cancer management include recommended timeframes for the intervals from one timepoint to another in the disease care pathway. British Thoracic Society guidelines [6] suggested the timeframe from diagnosis to treatment commencement should be a maximum of four weeks for chemotherapy, seven weeks for radiotherapy, and eight weeks for surgery. However, in Australia any definitive treatment (surgery, chemotherapy or radiotherapy) should be commenced within two weeks of diagnosis [7] and in Denmark, the recommendation is two weeks for surgery, 15 days for radiotherapy and 11 days for chemotherapy [8]. In the USA, recommendations are that treatment should commence within six weeks of diagnosis [9]. Despite recommended consensus of best practice for diagnosis

and treatment in guidelines, time from the onset of symptoms to diagnosis, on which prognosis is highly dependent, appears to vary widely in practice. A study in the USA found that the median interval from onset of symptom to diagnosis was 180 days [10], while a systematic review reporting studies from Canada, Sweden, France, Spain, and Norway found the time from symptom onset to initiation of treatment ranged widely from 47 to 138 days [11]. The same time span was 194 days in a study conducted in India [12].

Patients with lung cancer may present with a variety of symptoms which are not unique to that condition. Symptoms of lung cancer include haemoptysis, cough, breathlessness, hoarseness of voice, decreased appetite, weight loss, and fatigue. However, in recent years haemoptysis is reported less and cough and breathlessness are reported more as prominent symptoms [13]. The presence of comorbidities with similar symptoms can make the underlying cancer even more difficult to detect [14]. Sometimes, symptoms are misattributed to smoking or to comorbidities such as chronic obstructive pulmonary disease (COPD) or cardiac diseases, resulting in delayed care-seeking [15].

The majority of lung cancer guidelines have been developed and tested for well-resourced health systems and most of the studies on timeliness of seeking care of patients with lung cancer were conducted in developed countries. Little is known about the lung cancer journey among patients navigating health systems in low and middle income countries (LMIC), although a scoping review reported patients' reluctance in reporting symptoms, a shortage of pulmonary specialists, misdiagnosis, multiple physician consultations and long wait times due to a lack of capacity of health systems as contributing to longer intervals between symptom presentation and lung cancer diagnosis and care in LMICs [16].

Bangladesh is one of the most densely populated LMICs with a population of 163 million people [17] and a GDP per capita of US$ 1,827 [18]. Although only one fifth the size of its neighbour Pakistan in geographical area, it has just on 75% the population of that country. One-fifth (21.8%)

of the population live below the national poverty line [19] and the country has a large rural population of 65% [20]. In Bangladesh, there were over 250,000 premature deaths from noncommunicable diseases in 2018, 26% of which were due to cancer (108,000) [17]. Lung cancer is the most common cancer diagnosis in males in Bangladesh and is the commonest type of cancer-related deaths [21]. Most cases of lung cancer are diagnosed at an advanced stage when the options for treatment are limited [22].

The health system of Bangladesh is structured quite differently from those in Western countries which have informed current understanding of the lung cancer patient journey. There is no structured referral system from primary care to specialist care and there are no restrictions on patients seeking care from specialists without having appropriate referral from primary healthcare providers. Referral pathways are fragmented and there is no mechanism to know whether consumers have taken up referrals [23]. Bangladesh has less than one physician (0.53) per 1,000 people [24] and 7.9 hospital beds per 10,000 population, whereas in Denmark, the USA and Australia, the bed numbers are 26, 27.7 and 38.4 beds per 10,000, respectively [25]. Formal healthcare providers in the country include community clinics, health centres, district hospitals, public medical college hospitals and public specialised hospitals which are public healthcare providers. The other formal healthcare providers are the private medical college hospitals, private clinics, Non-Government Organization (NGO) hospitals, and NGO health workers in rural areas providing domiciliary visits. There is low utilisation of public health facilities in Bangladesh for a range of reasons including limited opening hours and lack of availability of medicines [26] and about 72% of healthcare expenses are paid out of pocket [27]. According to an article published in 2013, there are 19 hospitals in the country, mostly centrally located and providing regular cancer treatment [21]. There are an estimated 1.7 radiation oncologists per 10,000 cancer patients [17].

In LMICs, informal healthcare providers who lack recognised training comprise a significant portion of the health system, operating generally as individuals rather than via institution [28]. Pharmacies, traditional healthcare providers and village doctors are the common informal healthcare providers in Bangladesh [29]. Pharmacies, which might be more accurately described as retail medicine shops or drug sellers, are often the first point of contact for people in Bangladesh, specifically those from lower socioeconomic status [30]. They are popular for a range of reasons including close proximity to residence, easy accessibility of medicines without going through any investigation, bypassing consultation with qualified healthcare providers primarily due to bypassing out-of-pocket, unregulated, costly consultation fees, accessibility of cheap brands of medicines, pay later credit options, shorter waiting times and longer opening hours compared to public primary care facilities [31]. These pharmacies provide symptomatic treatment and advice on diseases and health conditions and dispense medication without a prescription, despite this being prohibited under the drug licence law [32]. Usually, a pharmacist learns about the specific treatment and management for a health condition by reviewing the prescriptions they receive from any qualified physician, which they apply for patients who bypass consultation with a physician. A country-wide survey of Bangladesh pharmacies revealed only 35% of customers had a prescription for the medication they received. The same survey showed that 51% of the pharmacies were staffed by personnel who had undertaken a three-month pharmacy course to enable them to run the retail shop and 70% of staff had 12 years or less of schooling [33]. In Bangladesh, traditional healthcare providers (ayurvedic, homeopathic, unanie/kabiraji - herbal medicine providers) are also common and popular in rural areas. These traditional healthcare providers have their own medicine preparations which they provide during their consultations. A study suggested that although traditional medicine is popular in both rural and urban areas, inadequate monitoring leads to improper preparation of medicines or even the manufacturing of medicines without legal permission, raising questions about their efficacy [26].

There is an overall lack of evidence in Bangladesh and across the subcontinent regarding timeliness of seeking care of lung cancer patients. For patients with lung cancer in Bangladesh, we currently do not know what symptoms trigger care-seeking, whether care-seeking occurs in a timely way, how long the intervals are between timepoints in the care-seeking pathway, or which healthcare providers are consulted along the lung cancer care pathway. Hence, the aims of this study were to (1) assess the symptom(s) that motivated patients to seek healthcare [triggering symptom(s)], (2) determine which healthcare providers the patients chose to seek healthcare from, and (3) measure the intervals between timepoints in the care-seeking pathway of patients with lung cancer in Bangladesh.

MATERIALS AND METHODS

Study design and population

A cross-sectional study was conducted in three study sites in Dhaka, Bangladesh. The sites included one public hospital, one university teaching hospital and one low cost philanthropic cancer hospital. The National Institute of Cancer Research and Hospital (NICRH) is a 300-bed tertiary level public hospital dedicated only for cancer care and provides the maximum load of cancer management in the country. Bangabandhu Sheikh Mujib Medical University (BSMMU) is a postgraduate medical institute with a limited 36-bed inpatient capacity oncology department which accepts both public and private referrals. Ahsania Mission Cancer & General Hospital (AMCGH) is the largest non-profit hospital in the country, currently having a 250-bed inpatient capacity dedicated for cancer care. Patients from across the country receive care from these three hospitals, however, anecdotal evidence suggests that AMCGH receives more patients from higher income groups than the other two hospitals.

Inpatients aged ≥18 years with a known diagnosis of lung cancer, who were able to understand the Participant Information and Consent Form and could nominate a family member or carer, if

needed, to provide information on their behalf were eligible to participate in the study. Potential participants were identified by doctors or nurses at the study sites from the inpatient files as per the inclusion criteria, after which the investigators approached eligible participants for written informed consent. Eligible participants were given information about the voluntary nature of their involvement in the study, details of the study and contact details of the investigators. The participant information was read to those participants who were illiterate. If the patient was feeling unwell and did not wish to provide answers themselves, patients were asked if they wanted to nominate caregivers to provide data on their behalf, and with consent from patients, caregivers were interviewed separately. Participants provided a signature as a sign of their consent or thumb impression for those who were illiterate.

Questionnaire and data collection procedure

A questionnaire was developed informed by relevant literature and knowledge of the local health system. Questions and response options were designed to be appropriate to the country context and study objectives. The questionnaire was developed in English and then translated into Bengali with the whole process going through 'translation and back translation' involving two of the investigators and two public health researchers, all of whom were bilingual. The Bengali version of the questionnaire was pilot-tested to ascertain consistency, appropriateness of language, and the best sequence of questions. The questionnaire was pilot-tested with eight lung cancer patients to ensure the questions were understandable and interpreted in a similar way by the participants. Based on the pilot test, several questions were rephrased for better understanding. Participants in the pilot testing were excluded from the final sample for data analysis. The questionnaire collected data under four sections 1) Socio-demographics such as age, sex, residence, education, marital status, type of family, and monthly household income, 2) History of illness 3) Symptoms information including date of onset of symptoms, first symptoms, symptoms that triggered healthcare seeking, 4) Help-seeking practice including choice of

healthcare provider for first contact, time to travel to healthcare provider, whether additional healthcare provider consulted before diagnosis, date of diagnosis, time to travel to diagnostic facility, date of referral for treatment, additional healthcare provider consulted after diagnosis before starting treatment, and date of initiation of treatment.

The structured questionnaire was administered face-to-face by three data collectors, who were medical doctors, and none was involved in providing treatment to the participants. Two were trained to conduct the survey by the first author who also participated in data collection. Information on different timepoints in the healthcare seeking pathway was collected from hospital clinical files, and from the patient-held clinical files to minimise recall bias. Permission was obtained from the hospital authority and informed consent was sought from the patients to examine both types of clinical files. After obtaining informed consent, a participant's interview was done at the bedside if privacy could be maintained and, in the case of the caregiver, the interview was conducted in the duty doctor's room in private. In addition to asking about different timepoints of care seeking in the disease cycle as part of the questionnaire, data were extracted from patient-held clinical files available at the time of interview. In Bangladesh, patients keep a hard-copy record of their previous treatment documents which includes previous prescriptions, investigation reports and discharge summary of previous hospital admission. They generally have this with them when receiving care in a hospital. This process served as cross-checking to minimise recall bias. Where there was a mismatch of information provided by the participant and that extracted from hospital documents, the dates derived from the hospital were accepted. To ensure data quality, the first author monitored administration of the questionnaire throughout the data collection period. Five percent of the collected data were reviewed to check the accuracy and completeness of the data. No discrepancies were found in the data.

Measurement of healthcare seeking intervals

Date variables: (i) date of first symptom, (ii) date of first contact with any healthcare provider, (iii) date of diagnosis, (iv) date of referral for treatment and (v) date of treatment initiation.

Time Intervals:

Time intervals were estimated by calculating the duration (days) from one time point to another. The time interval was recorded as '0' days if the participant contacted a healthcare provider on the first day the symptoms appeared; was diagnosed on the day of first healthcare provider contact; was referred at the time of receiving the diagnosis and started treatment on the day of referral. Seven time intervals were estimated. The operational definitions were as follows:

- First interval (Appraisal & Help Seeking) was defined as the time between the date of onset of symptoms and the date of the first contact with any healthcare provider, including informal healthcare providers
- Second interval (Diagnosis) was defined as the time between the date of the first contact with a healthcare provider and the date of the confirmed diagnosis
- Third interval (Referral) was defined as the time between the date of the confirmed diagnosis and the date of the referral for treatment
- Fourth interval (Treatment) was defined as the time between the date of the referral and the date of the initiation of treatment
- Symptom to diagnosis interval was defined as the time between the date of the first symptom appearance to the date of the confirmation of diagnosis
- Diagnosis to treatment interval was defined as the time between the date of the confirmation of diagnosis and the date of the initiation of treatment
- Symptom to treatment interval was defined as the time between the date of the symptom appearance and the date of the initiation of treatment

Data analysis

Descriptive analyses were performed using frequency, mean and standard deviation (SD), median and interquartile range (IQR) depending on the distribution of the data. Comparison of sociodemographic characteristics and type of healthcare providers for first contact (formal and informal healthcare provider) were performed using chi-square analysis; Wilcoxon Rank Sum test was done to compare intervals with type of healthcare providers first contacted, and significance was set at p-value <0.05. The data were analysed using SPSS (version 25).

Ethics

This study obtained ethical approval from the University Human Ethics Committee of La Trobe University, Melbourne, Australia (HEC19154); National Institute of Cancer Research & Hospital, Dhaka, Bangladesh (NICRH/Ethics/2019/504); and Ahsania Mission Cancer and General Hospital, Dhaka Bangladesh (DAM/AMCGH/1900-2019-2063).

RESULTS

A total of 418 questionnaires were completed between 10 October 2019 and 13 February 2020, of which 318 were with the patients and 100 were with the patients' nominated caregivers. Of 424 eligible patients, only six refused to participate, with 285 participants from NICRH, 27 from BSMMU and 106 from AMCGH were included in the study. The demographic characteristics of the respondents from each site are described in Table 1. The mean age of the sample was 57 years (SD ±9.86 with a range of 25-82 years). The majority of patients were over 55 years old (68%), with less than 10% aged under 45 years. Participants were predominantly from rural area (88%) and the majority of participants were illiterate (64%). The mean year of education was 4 years (SD ±5 and range 0-17 years). The median monthly household income was BDT 15500 (USD 182). The lowest quartile had an income of BDT 10000 and below, whereas the upper

quartile had an income of BDT 50000 and above. The demographic characteristics of the samples are almost similar across the study sites.

Table 1: Characteristics of the participants (n = 418)

	NICRH (n, %)	BSMMU (n, %)	AMCGH (n, %)	Total (n, %)
**Age Group (in years)**				
<35	7 (2.5)	0	1 (0.9)	8 (1.9)
35-44	20 (7)	4 (14.8)	5 (4.7)	29 (6.9)
45-54	65 (22.8)	2 (7.4)	29 (27.4)	96 (23)
55-64	120 (42.1)	12 (44.4)	45 (42.5)	177 (42.3)
≥65	73 (25.6)	9 (33.3)	26 (24.5)	108 (25.8)
**Sex**				
Male	260 (91.2)	23 (85.2)	92 (86.8)	375 (89.7)
Female	25 (8.8)	4 (14.8)	14 (13.2)	43 (10.3)
**Marital Status**				
Married	266 (93.3)	25 (92.6)	97 (91.5)	388 (92.8)
Not Married	19 (6.7)	2 (7.4)	9 (8.5)	30 (7.2)
**Area of Residence**				
Urban	34 (11.9)	2 (7.4)	14 (13.2)	50 (12)
Rural	251 (88.1)	25 (92.6)	92 (86.8)	368 (88)
**Family Structure**				
Nuclear family	120 (42.1)	10 (37.0)	34 (32.1)	164 (39.2)
Joint/extended family	165 (57.9)	17 (63.0)	72 (67.9)	254 (60.8)
**Highest Education Level**				
Illiterate & below primary education	196 (68.8)	21 (77.8)	52 (51.1)	269 (64.4)
Primary	48 (16.8)	3 (11.1)	13 (12.3)	64 (15.3)
Secondary	24 (8.4)	1 (3.7)	14 (13.2)	39 (9.3)
Higher Secondary	7 (2.5)	2 (7.4)	9 (8.5)	18 (4.3)
Bachelor's degree & above qualification	10 (3.5)	0	18 (17)	28 (6.7)
**Monthly Household Income**				
BDT ≤15000 (≤US$176)	190 (66.7)	13 (48.1)	6 (5.7)	209 (50)
BDT 15001 – 50000 (US$176-588)	80 (28.1)	13 (48.1)	33 (31.1)	126 (30.1)
BDT 50001 – 100000 (US$588-1176)	13 (4.6)	0	38 (35.8)	51 (12.2)
BDT ≥100001 (≥US$1176)	2 (0.7)	1 (3.7)	29 (27.4)	32 (7.7)
**Travel time to first healthcare provider**				
<10 min	91 (31.9)	8 (29.6)	43 (40.6)	142 (34)
10 to 30 min	125 (43.9)	11 (40.7)	24 (22.6)	160 (38.3)
31 to 60 min	43 (15.1)	4 (14.8)	25 (23.6)	72 (17.2)
61 min or more	26 (9.1)	4 (14.8)	14 (13.2)	44 (10.5)

*1 US$ = 85 BDT (Bangladeshi Taka), NICRH - National Institute of Cancer Research and Hospital, BSMMU - Bangabandhu Sheikh Mujib Medical University, AMCGH - Ahsania Mission Cancer and General Hospital

Table 2 shows the first symptom(s) experienced and the symptoms that led to respondents seeking healthcare. Half of the participants (226, 54%) mentioned just one triggering symptom. However, 46% of the participants reported a combination of symptoms triggering healthcare seeking. At least two symptoms triggered healthcare-seeking in 34% of participants, while 11% of participants mentioned three or four symptoms as their triggering factor. Table 3 shows the most frequent single triggering symptom and most common combination of triggering symptoms.

Table 2: First symptoms and triggering symptoms to seek healthcare provider (n = 418)

	Symptoms first noticed		Triggering symptoms	
	Frequency*	%	Frequency*	%
Cough	369	88.28	275	65.79
Chest pain	236	56.5	162	38.76
Shortness of breath	108	25.84	68	16.27
Haemoptysis	103	24.64	62	14.83
Lack of appetite	43	10.29	19	4.55
Hoarseness of voice	40	9.57	19	4.55
Weight loss	10	2.39	2	0.48
Fatigue	6	1.44	5	1.20
Persistent or recurrent infections	2	0.48	0	0
Other uncommon symptoms	65	15.6	51	12.20

*as multiple triggering symptoms were reported by a single respondent, the total number is more than 418. % are calculated as a proportion of the total sample.

Table 3: Most common combination of triggering symptoms

	Frequency*	%
Cough as only triggering symptom	95	22.72
Cough and chest pain	91	21.77
Cough and shortness of breath	54	12.92
Cough and haemoptysis	37	8.85
Chest pain and shortness of breath	22	5.26
Cough and hoarseness of voice	15	3.59
Chest pain and hoarseness of voice	15	3.59
Chest pain and haemoptysis	14	3.35

The majority of participants (60%) had contact with an informal healthcare provider (pharmacy 43%, village doctor or traditional healer 17%) as their first point of contact, whilst 22% went to a General Practitioner (GP) and the remainder to other formal healthcare providers (Table 4). Only 8% of the participants sought care from a single provider before undergoing confirmation of a lung cancer diagnosis of lung cancer. The majority (92%) went to multiple healthcare providers before getting confirmation of their lung cancer diagnosis. Two-fifths consulted two additional healthcare providers (42%), one quarter consulted one additional healthcare provider and 81 (19%) respondents consulted three additional healthcare providers pre-diagnosis. After first contact, most of the additional pre-diagnosis care seeking was with public healthcare providers (76%) and specialists (48%) (Table 4).

Table 4: Contact with healthcare providers at different timepoints

Healthcare providers	First point of contact	Additional pre-diagnosis	Additional pre-treatment
	Frequency N=418 (%)	Frequency N=383* (%)	Frequency N=96* (%)
Public Healthcare	28 (6.7)	319 (76.3)	34 (8.1)
Private and NGO Healthcare	27 (6.5)	119 (28.5)	47 (11.2)
GPs (MBBS)	91 (21.8)	123 (29.4)	15 (3.6)

Healthcare providers	First point of contact	Additional pre-diagnosis	Additional pre-treatment
	Frequency N=418 (%)	Frequency N=383* (%)	Frequency N=96* (%)
Specialist Doctors	22 (5.3)	200 (47.9)	35 (8.4)
Pharmacy (over the counter)	179 (42.8)	7 (1.7)	0
Village Doctor & Traditional Medicine	71 (17)	17 (4.1)	5 (1.2)

* Multiple responses were allowed.

Once a diagnosis of lung cancer was confirmed by a formal healthcare provider, almost one-quarter of participants consulted at least one additional healthcare provider before starting treatment. In Bangladesh, specialist doctors provide healthcare in private hospitals and NGO hospitals. Private and NGO healthcare providers were consulted by 47 (11% of 418) participants, 35 (8% of 418) consulted additional specialist doctors and 34 (8% of 418) consulted in public healthcare facilities. Notably, consulting with traditional medicine or village doctors was less frequent compared to first point of contact (17%), at additional pre-diagnosis consultation (4%) and additional pre-treatment consultation (1%) timepoints (Table 4).

At the first point of contact, the majority of participants in all age groups (60% overall) visited informal healthcare providers which included pharmacies or drug sellers, village doctors and traditional medicine practitioners compared to formal healthcare providers i.e., medically trained personnel. Rural participants were more likely to visit informal providers (61%) and just over half (54%) of the urban participants went to formal healthcare providers as their first point of contact. Most of the participants with little or no education went to informal healthcare providers (67%), and participants with bachelor degree or higher visited formal healthcare providers (71%). Participants with higher income went to formal healthcare providers as their first contact (67%), while 69% of the lower income group went to informal healthcare providers. There were significant differences between first contact with a provider and area of residence, education, and monthly income (p-value <0.05) (Table 5).

Table 5: First healthcare contact according to sociodemographic characteristics (n=418)

	Formal healthcare provider (n, %)	Informal healthcare provider (n, %)	p-value
**Age Group (in years)**			
<35	3 (37.5)	5 (62.5)	0.40
35-44	11 (37.9)	18 (62.1)	
45-54	31 (32.3)	65 (67.7)	
55-64	79 (44.6)	98 (55.4)	
≥65	44 (40.7)	64 (59.3)	
**Sex**			
Male	152 (40.5)	223 (59.5)	0.67
Female	16 (37.2)	27 (62.8)	
**Marital Status**			
Married	156 (40.2)	232 (59.8)	098
Not Married	12 (40.0)	18 (60.0)	
**Family Structure**			
Nuclear family	70 (42.7)	94 (57.3)	0.40
Joint/extended family	98 (38.6)	156 (61.4)	
**Area of Residence**			
Urban	27 (54.0)	23 (46.0)	0.03
Rural	141 (38.3)	227 (61.7)	
**Highest Education Level**			
Illiterate & below primary education	89 (33.1)	180 (66.9)	<0.001
Primary education	28 (43.8)	36 (56.3)	
Secondary & higher secondary education	31 (54.4)	26 (45.6)	
Bachelor's & above qualification	20 (71.4)	8 (28.6)	
**Monthly Household Income**			
BDT ≤15000 (≤US$176)	65 (31.1)	144 (68.9)	<0.001
BDT 15001 – 50000 (US$176-588)	54 (42.9)	72 (57.1)	
BDT 50001 – 100000 (US$588-1176)	28 (54.9)	23 (45.1)	
BDT ≥100001 (≥US$1176)	21 (65.6)	11 (34.4)	

The median duration of intervals between first contact with provider and diagnosis, onset of symptoms to diagnosis, and onset of symptoms to initiation of treatment were significantly higher among patients who sought care from informal providers as their first point of contact compared to formal healthcare providers (p value <0.05). However, the median duration of interval between onset of symptoms and first contact with provider was shorter in those who visited informal providers compared to formal healthcare providers (p value <0.05) (Table 6).

Table 6: Duration of intervals in care seeking with first point of contact*

	Formal healthcare provider Median	Informal healthcare provider Median	p-value
Duration between onset of symptoms and first contact with provider	13	10	0.03
Duration between first contact with provider and diagnosis	89	121	<0.001
Duration between onset of symptoms and diagnosis	104	136	<0.001
Total duration from onset of symptoms to initiation of treatment	131	171	<0.001

*Not all the intervals are related with the first contact; the most relevant intervals were examined.

The entire care pathway was segmented in seven intervals. The median time between onset of symptom and initiation of treatment was 151 days. As a segment, the longest duration in the care seeking pathway was the duration from symptom to diagnosis (median 121 days) and the second longest duration was from first contact with any healthcare provider to diagnosis (median 107 days). The shortest duration was observed between diagnosis and referral for treatment (median 3 days) and onset of symptom to first contact with any healthcare provider (median 10 days) (Figure 1).

Figure 1: Timepoints and intervals in the care seeking pathway for lung cancer

DISCUSSION

This study is the first to investigate the care trajectory and duration of intervals in the care seeking pathway for lung cancer in Bangladesh. The mean total duration from onset of symptoms to initiation of treatment for our study was 186.4 ± 140.4 days with a median of 151 days (13 – 1460 days). This is longer than the duration reported in a study conducted in India, a neighbouring country, where the median duration was 126 days [34]. The total duration in our study, from onset of symptom(s) to initiation of treatment is also longer in comparison to similar studies conducted in western countries such as USA (median of 52 days) [35], Greece (median of 81 days) [36], and Turkey (median 57 days) [11]. These findings suggest that there may be weaknesses in the health system and the way care is currently provided to patients with lung cancer in Bangladesh, as well as individual factors that could be contributing to unacceptable delays.

Some of the intervals appeared to be shorter than those observed in studies from other countries. The mean duration between onset of symptoms and first care seeking was 15.2 ± 24.4 (median 10) days in this study. This is shorter than time periods reported from symptom onset to contact with a medical doctor in other countries, including 71 days in Nepal [37], 94 days in India [16], and 35 days or 49.9 ± 96.9 days in Turkey [11, 38]. In this study, informal healthcare providers

were the first choice for seeking healthcare reported by most respondents (60%), hence this duration is shorter than other studies mentioned above. Studies conducted in LMICs have reported that, closer vicinity, easier access, and affordability are the main reasons that patients seek care from informal healthcare providers compared to formal healthcare providers [28]. Pharmacies in Bangladesh are not typically staffed by tertiary trained health professionals, operating rather as medicine retailers. Thus, the most accessible form of healthcare in Bangladesh is provided by people with very limited training in symptomatology, knowledge of basic primary healthcare, drugs, and their administration. Other research in Bangladesh has reported that people will self-medicate or use local remedies before seeking healthcare from a medically trained professional [39]. In a study on acute respiratory illness 62% of people who had cough, fever, running nose, myalgia, or a sore throat sought to treat themselves and pharmacies were the first point of contact for 90% of the respondents [40].

The period from onset of symptoms to confirmed diagnosis was longer than in other countries where similar studies were conducted. In our study the mean duration between onset of symptoms and diagnosis was 151 ± 132 days with a median of 121 days. This is longer compared to studies conducted in India (median of 107 days) [34], Greece (median of 52 days) [36], Turkey (median of 49 days) [11], and Canada (median of 90 days) [41]. As noted above, this difference may reflect the inclusion of pharmacies as the first healthcare provider. This is supported by the finding that the mean duration in our study from first contact with any healthcare provider to diagnosis was longer than other studies (135.8 ± 126.7 days). In Nepal, this step took 50 days on average [37] where the first contact was a medically trained provider. This interval involves the patient reaching qualified doctors and the hospital system, and suggests that the sooner the patient gets diagnosed, the sooner the treatment initiates. In order to ensure early diagnosis, it is paramount that patient’s healthcare seeking should start from medically trained provider rather than informal healthcare.

It is noteworthy to mention that the delay occurred in the early phase of the disease care pathway, mostly in the pre-diagnosis phase, and after diagnosis the intervals became shorter compared to pre-diagnosis intervals. The mean interval from diagnosis to initiation of treatment was 35.3 ± 42.9 days with a median of 22 days in this study. Although it was not within the acceptable maximum timeframe for diagnosis to initiation of treatment described in guidelines in Australia, Denmark and Sweden (14 days), it was within an acceptable range for guidelines in the USA (42 days) and the UK (48 days). Further, the duration (diagnosis to initiation of treatment) in this study was shorter than the same interval reported in the USA (median of 27 days) [42] and mean of 24.4 ± 54.9 days) [38] and was within the range for medians of 6-45 days reported by Jacobsen et al [43].

The pattern in the duration of intervals between key timepoints in the care seeking pathway for lung cancer in this study suggests that the reason for the duration from onset of symptoms to initiation of treatment was long as care seeking mostly started from informal healthcare in Bangladesh. There were significant differences in timepoints according to whether first contact was with informal healthcare providers compared with formal. While the median duration from onset of symptoms to first contact was significantly shorter for those who used informal providers, intervals between onset of symptoms and diagnosis and treatment were significantly longer for people who sought help from informal providers. Moreover, those who sought care from informal providers were more likely from rural areas, had fewer years of education and a lower income. Similar findings were reported in other studies conducted in Bangladesh [44, 45] and India [46]. A study reporting reasons to use over the counter medicines in India and Nepal identified financial constraints, non-availibility of health professionals and increased health care cost to be the major cause [47]. This might be a possible explanation of the finidng in our study as the majority of respondents sought care from pharmacies as their first contact. In addition, traditional healing practice is very widely used in rural Bangladesh, with 75-80% of the rural population dependent

on it in the absence of access to formal healthcare [45]. However, only 17% of study participants in this study reported using of traditional helaers for management of lung cancer. The use of traditional medicine is deeply embedded in the health belief system of the rural population in Bangladesh [44]. Reliance on traditional health practices poses a threat for timely initiation of proper medical care for patients with cancer which is also reported in studies conducted in Bangladesh [48], India [49] and Malaysia [50]. So, while time to seek and receive this care is short, it is unlikely to be effective for someone with lung cancer.

This study suggests the need to raise awareness of the importance of seeking healthcare from medically trained healthcare professionals for lung cancer symtpoms. Efforts should be given to creating mass awareness campaigns on recognising ‘danger signs’ for lung cancer as well as strengthening health systems to provide ‘fast-track’ access to formal healthcare providers for any suspected case of lung cancer for early diagnosis and management. Informal healthcare providers should be trained to recognise lung cancer symptoms and to refer suspected patients early to formal healthcare providers; introducing a reward system could encourage adoption of such practice. Further research will be needed to explore factors associated with longer and shorter durations in the health seeking pathway and their effects on lung cancer survival in Bangladesh.

This study used data from patients in three urban hospitals in Bangladesh, so findings did not represent the experiences of people receiving treatment for lung cancer in the entire country. However, the included hospitals were the three key referral hospitals for cancer management in Bangladesh and the sample included patients from all over the country and from all socioeconomic status. It also did not capture the experience of anyone who did not receive treatment from a hospital.

CONCLUSION

This paper is the first to describe the lung cancer patient journey in Bangladesh. The study suggests that the patterns in the average duration between timepoints in healthcare seeking from onset of symptoms to initiation of treatment in lung cancer care in Bangladesh are different to those in other countries, being shorter at some points and longer at others. Informal care was convenient and easy to access, which resulted in a shorter interval between symptom(s) and first contact compared to the formal pathway. However, the quicker access to informal care did not translate into quicker provision of lung cancer treatment and led to significant delays in initiation of treatment. To ensure timely lung cancer diagnosis and care, there is a need to better understand the reasons for the intervals between care points observed in this study. This may help to develop health system specific and clinically evidence-based benchmarks for Bangladesh.

Acknowledgement

We would like to thank the participants, doctors, nurses and data collectors for their time, patience and support. We also thank Professor Dr. Sarwar Alam and Associate Professor Dr. Sadia Sharmin of BSMMU, Professor Dr. Md. Mahbubur Rahman of NICRH, Professor Dr. Qamaruzzaman Chowdhury and Dr. Nahid Sultana of AMCGH for their support in implementing the study in Bangladesh.

Funding

This research received no specific grant from any funding agency in the public, commercial or not-for-profit sectors.

REFERENCE

1. World Cancer Report: Cancer Research for Cancer Prevention. Lyon, France: International Agency for Research on Cancer, WHO; 2020. Available from: http://publications.iarc.fr/586.

2. SEER Cancer Statistics Review, 1975-2015 Bethesda, MD: National Cancer Institute; 2018 [Available from: https://seer.cancer.gov/archive/csr/1975_2015/.
3. Alanen V, Koivunen JP. Association of diagnostic delays to survival in lung cancer: single center experience. Acta Oncol. 2019;58(7):1056-61.
4. Allemani C, Weir HK, Carreira H, Harewood R, Spika D, Wang XS, et al. Global surveillance of cancer survival 1995-2009: analysis of individual data for 25,676,887 patients from 279 population-based registries in 67 countries (CONCORD-2). Lancet (London, England). 2015;385(9972):977-1010.
5. Deffebach ME, Humphrey L. Lung Cancer Screening. Surg Clin North Am. 2015;95(5):967-78.
6. BTS recommendations to respiratory physicians for organising the care of patients with lung cancer. The Lung Cancer Working Party of the British Thoracic Society Standards of Care Committee. Thorax. 1998;53 Suppl 1(Suppl 1):S1-8.
7. Stirling RG, Evans SM, McLaughlin P, Senthuren M, Millar J, Gooi J, et al. The Victorian Lung Cancer Registry pilot: improving the quality of lung cancer care through the use of a disease quality registry. Lung. 2014;192(5):749-58.
8. Christensen NL, Jekunen A, Heinonen S, Dalton SO, Rasmussen TR. Lung cancer guidelines in Sweden, Denmark, Norway and Finland: a comparison. Acta Oncol. 2017;56(7):943-8.
9. Asch SM, Kerr EA, Hamilton EG, Reifel JL, McGlynn EA. Quality of Care for Oncologic Conditions and HIV: A Review of the Literature and Quality Indicators: RAND Corporation; 2000.
10. Koo MM, Zhou Y, Lyratzopoulos G. Delays in diagnosis and treatment of lung cancer: Lessons from US healthcare settings. Cancer epidemiol. 2015;39(6):1145-7.
11. Vinas F, Ben Hassen I, Jabot L, Monnet I, Chouaid C. Delays for diagnosis and treatment of lung cancers: a systematic review. Clin Respir J. 2016;10(3):267-71.
12. Dwivedi AK, Dwivedi SN, Deo S, Shukla R, Pandey A, Dwivedi DK. An epidemiological study on delay in treatment initiation of cancer patients. Health. 2012;04(02):66-79.
13. Chowienczyk S, Price S, Hamilton W. Changes in the presenting symptoms of lung cancer from 2000-2017: a serial cross-sectional study of observational records in UK primary care. Br J Gen Pract. 2020;70(692):e193-e9.
14. Neal RD, Robbé IJ, Lewis M, Williamson I, Hanson J. The complexity and difficulty of diagnosing lung cancer: findings from a national primary-care study in Wales. Prim Health Care Res Dev. 2015;16(5):436-49.
15. Brousselle A, Breton M, Benhadj L, Tremblay D, Provost S, Roberge D, et al. Explaining time elapsed prior to cancer diagnosis: patients' perspectives. BMC Health Serv Res. 2017;17(1):448.
16. Nwagbara UI, Ginindza TG, Hlongwana KW. Health systems influence on the pathways of care for lung cancer in low- and middle-income countries: a scoping review. Global Health. 2020;16(1):23.
17. WHO. Cancer Country profiles 2020. International Cancer Control Partnership (ICCP); 2020.
18. MOF. Bangladesh Economic Review Dhaka, Bangladesh: Finance Division, Ministry of Finance, Government of Bangladesh; 2019 [updated June 2 2020. Available from: https://mof.portal.gov.bd/site/page/28ba57f5-59ff-4426-970a-bf014242179e/Bangladesh-Economic-Review.
19. Bank AD. Poverty Data: Bangladesh: Asian Development Bank; 2020 [Available from: https://www.adb.org/countries/bangladesh/poverty#:~:text=In%20Bangladesh%2C%20the%20population%20living,2016%20to%209.2%25%20in%202019.
20. Bank TW. Rural population (% of total population) - Bangladesh: The World Bank; 2020 [Available from: https://data.worldbank.org/indicator/SP.RUR.TOTL.ZS?locations=BD.

21. Hussain SM. Comprehensive update on cancer scenario of Bangladesh. South Asian J Cancer. 2013;2(4):279-84.
22. Ahmed K, Emran AA, Jesmin T, Mukti RF, Rahman MZ, Ahmed F. Early detection of lung cancer risk using data mining. Asian Pac J Cancer Prev. 2013;14(1):595-8.
23. WHO. Bangladesh health system review: Manila : WHO Regional Office for the Western Pacific; 2015.
24. CIA. South Asia Bangladesh — The World Factbook - Central Intelligence Agency 2020 [updated June 29 2020. Available from: https://www.cia.gov/library/publications/the-world-factbook/geos/bg.html.
25. World Health Organization. Global Health Observatory data repository 2020 [Available from: https://www.who.int/data/gho.
26. Huque R. The Use of Traditional Medicine: A study in Bangladesh. Geneva Health Forum Archive [Internet]. 2014 25.06.2020. Available from: http://ghf.g2hp.net/2014/02/25/the-use-of-traditional-medicine-a-study-in-bangladesh/.
27. World Health Organization. Regional Office for South-East A. Health financing profile 2017: Bangladesh. New Delhi: World Health Organization. Regional Office for South-East Asia; 2017 2017.
28. Sudhinaraset M, Ingram M, Lofthouse HK, Montagu D. What is the role of informal healthcare providers in developing countries? A systematic review. PLoS ONE. 2013;8(2):e54978.
29. Ahmed SM, Evans TG, Standing H, Mahmud S. Harnessing pluralism for better health in Bangladesh. Lancet (London, England). 2013;382(9906):1746-55.
30. Ahmed SM, Hossain MA, Rajachowdhury AM, Bhuiya AU. The health workforce crisis in Bangladesh: shortage, inappropriate skill-mix and inequitable distribution. Hum Resour Health. 2011;9:3.
31. Saha S, Hossain MT. Evaluation of medicines dispensing pattern of private pharmacies in Rajshahi, Bangladesh. BMC Health Serv Res. 2017;17(1):136.
32. Rakib A, Sarwar S, Zannah S, Khanum S, Rashid M. A Survey of the Role of Community Pharmacists in Dhaka city, Bangladesh. Bangladesh Pharmaceutical Journal. 2015;18:137-41.
33. SIAPS. Baseline Study of Private Drug Shops in Bangladesh: Findings and Recommendations. Arlington, VA: Systems for Improved Access to Pharmaceuticals and Services, Center for Pharmaceutical Management; 2015.
34. Vashistha V, Choudhari C, Garg A, Gupta A, Parthasarathy G, Jain D, et al. The time required to diagnose and treat lung Cancer in Delhi, India: an updated experience of a public referral center. Applied Cancer Research. 2019;39(1).
35. Vidaver RM, Shershneva MB, Hetzel SJ, Holden TR, Campbell TC. Typical Time to Treatment of Patients With Lung Cancer in a Multisite, US-Based Study. J Oncol Pract. 2016;12(6):e643-53.
36. Kourlaba G, Gkiozos I, Kokkotou E, Stefanou G, Papaspiliou A, Syrigos K. Lung cancer patients' journey from first symptom to treatment: Results from a Greek registry. Cancer epidemiol. 2019;60:193-200.
37. Thapa B, Sayami P. Low lung cancer resection rates in a tertiary level thoracic center in Nepal--where lies our problem? Asian Pac J Cancer Prev. 2014;15(1):175-8.
38. Yurdakul AS, Kocatürk C, Bayiz H, Gürsoy S, Bircan A, Özcan A, et al. Patient and physician delay in the diagnosis and treatment of non-small cell lung cancer in Turkey. Cancer epidemiol. 2015;39(2):216-21.
39. Ahmed SM, Adams AM, Chowdhury M, Bhuiya A. Gender, socioeconomic development and health-seeking behaviour in Bangladesh. Soc Sci Med. 2000;51(3):361-71.
40. Chowdhury F, Sturm-Ramirez K, Mamun AA, Iuliano AD, Bhuiyan MU, Chisti MJ, et al. Factors driving customers to seek health care from pharmacies for acute respiratory illness and

treatment recommendations from drug sellers in Dhaka city, Bangladesh. Patient Prefer Adherence. 2017;11:479-86.
41. Wai ES, Mackinnon M, Hooker R, Moccia P, Perry KR, Truong PT. Wait times in diagnostic evaluation and treatment for patients with stage III non-small cell lung cancer in British Columbia. Am J Clin Oncol. 2012;35(4):373-7.
42. Gomez DR, Liao KP, Swisher SG, Blumenschein GR, Erasmus JJ, Jr., Buchholz TA, et al. Time to treatment as a quality metric in lung cancer: Staging studies, time to treatment, and patient survival. Radiother Oncol. 2015;115(2):257-63.
43. Jacobsen MM, Silverstein SC, Quinn M, Waterston LB, Thomas CA, Benneyan JC, et al. Timeliness of access to lung cancer diagnosis and treatment: A scoping literature review. Lung Cancer. 2017;112:156-64.
44. Haque MA, Louis VR, Phalkey R, Sauerborn R. Use of traditional medicines to cope with climate-sensitive diseases in a resource poor setting in Bangladesh. BMC Public Health. 2014;14:202.
45. Haque MI, Chowdhury A, Shahjahan M, Harun MGD. Traditional healing practices in rural Bangladesh: a qualitative investigation. BMC Complement Altern Med. 2018;18(1):62.
46. Panda A, Pradhan S, Mohapatro G, Kshatri JS. Predictors of over-the-counter medication: A cross-sectional Indian study. Perspect Clin Res. 2017;8(2):79-84.
47. Marathe PA, Kamat SK, Tripathi RK, Raut SB, Khatri NP. Over-the-counter medicines: Global perspective and Indian scenario. J Postgrad Med. 2020;66(1):28-34.
48. Akhtar K, Akhtar K, Rahman MM. Use of Alternative Medicine Is Delaying Health-Seeking Behavior by Bangladeshi Breast Cancer Patients. Eur J Breast Health. 2018;14(3):166-72.
49. Broom A, Doron A. The rise of cancer in urban India: Cultural understandings, structural inequalities and the emergence of the clinic. Health (London). 2012;16(3):250-66.
50. Merriam S, Muhamad M. Roles traditional healers play in cancer treatment in Malaysia: implications for health promotion and education. Asian Pac J Cancer Prev. 2013;14(6):3593-601.

Click here to access/download;Figure;Figure 1 Timepoints and intervals.tif

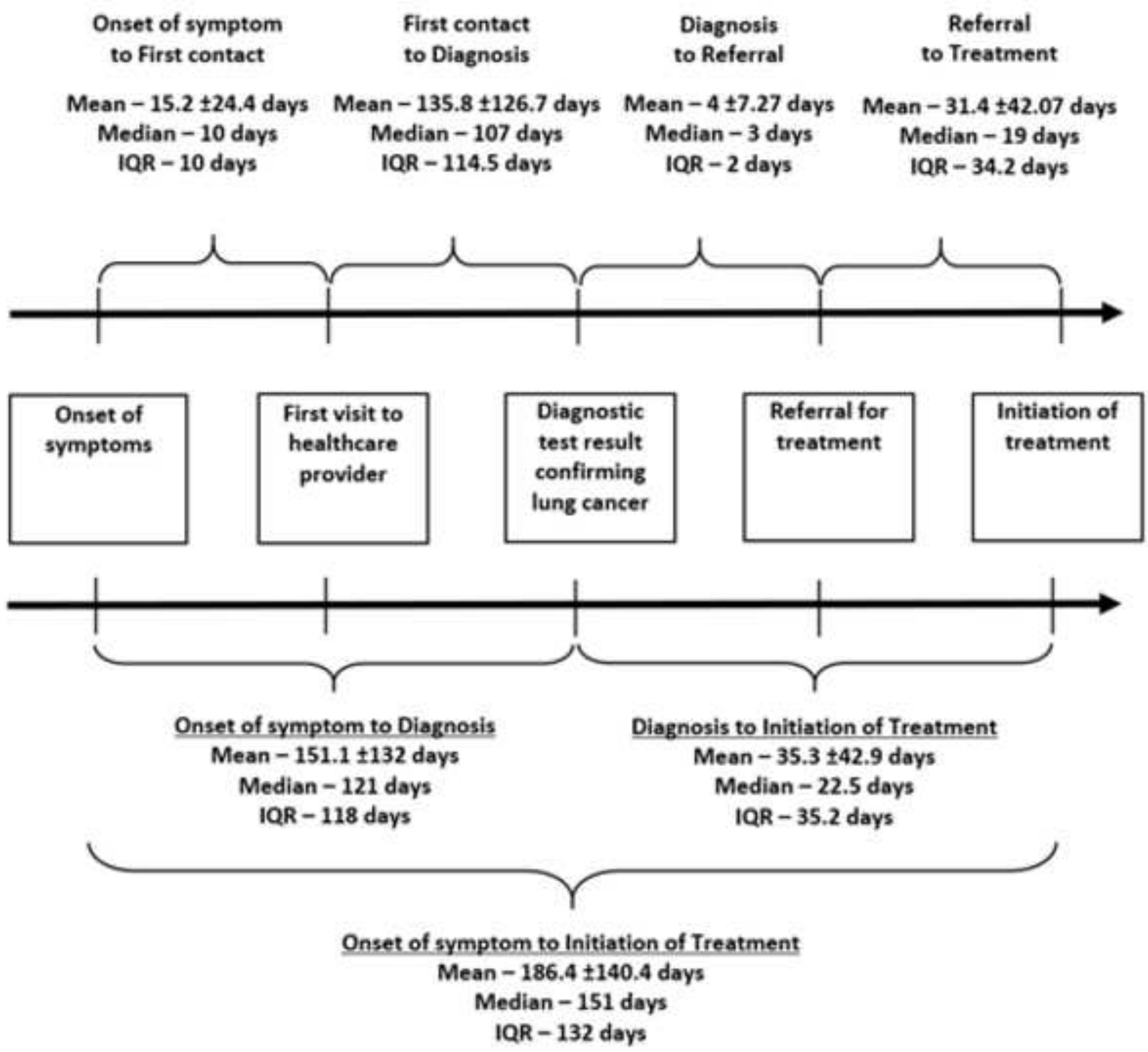

Supplement: Attachment — Submitted filename: PONE-D-20-39263_reviewer (2).pdf [file pone.0257301.s003.pdf]
