# Supplementary material for: Duration of intervals in the care seeking pathway for lung cancer in Bangladesh: A journey from symptoms triggering consultation to receipt of treatment
Source: PLoS One. 2021 Sep 10;16(9):e0257301. doi: 10.1371/journal.pone.0257301 (PMC8432814; doi:10.1371/journal.pone.0257301)
Supplement: Attachment — Submitted filename: Response to Reviewers.docx [file pone.0257301.s002.docx]

PONE-D-20-39263 Response to reviewers’ comments:

NOTE That page and line numbers refer to the clean version of the Revised Manuscript (without tracked changes)

|  | **Feedback** | **Response** |
| --- | --- | --- |
|  | **Journal Requirements:** |  |
| 1. | Please ensure that your manuscript meets PLOS ONE's style requirements, including those for file naming. | PLOS ONE style has been adopted for the manuscript. |
| 2. | Please provide further details on sample size and power calculations. | Details on sample size and power calculation are provided in p. 7, line 148 – 152. |
| 3. | In statistical methods, please refer to any post-hoc corrections to correct for multiple comparisons during your statistical analyses. If these were not performed, please justify the reasons. | Text added (p. 12, line 257-261) |
| 4. | We note that you have indicated that data from this study are available upon request. PLOS only allows data to be available upon request if there are legal or ethical restrictions on sharing data publicly. For information on unacceptable data access restrictions, please see http://journals.plos.org/plosone/s/data-availability#loc-unacceptable-data-accessrestrictions. | The data that support this study cannot be shared publicly because of privacy issues; data include identifiable personal health information. Data files will be available upon reasonable request to Professor Virginia Lewis and on the condition that the La Trobe University ethics committee grants permission for researchers who meet the criteria for access to confidential data. Contact information: [V.Lewis@latrobe.edu.au](mailto:V.Lewis@latrobe.edu.au) or directly to Human Research Ethics Committee: [humanethics@latrobe.edu.au](mailto:humanethics@latrobe.edu.au) |
|  | **In your revised cover letter, please address the following prompts:** |  |
| 5. | a) If there are ethical or legal restrictions on sharing a de-identified data set, please explain them in detail (e.g., data contain potentially identifying or sensitive patient information) and who has imposed them (e.g., an ethics committee). Please also provide contact information for a data access committee, ethics committee, or other institutional body to which data requests may be sent. | Added in revised cover letter as advised. See response to 4. above |
| 6. | If there are no restrictions, please upload the minimal anonymized data set necessary to replicate your study findings as either Supporting Information files or to a stable, public repository and provide us with the relevant URLs, DOIs, or accession numbers. Please see http://www.bmj.com/content/340/bmj.c181.long for guidelines on how to de-identify and prepare clinical data for publication. For a list of acceptable repositories, please see http://journals.plos.org/plosone/s/data-availability#loc-recommended-repositories. | As noted – there are restrictions on access to data. This has been added in the revised cover letter as advised. See response to 4. above |
|  | **Reviewer #1** |  |
| 7. | The introduction is too long, reads like a review article and includes information that could be summarized or is already provided in the discussion. Alternatively, some of this could be briefly summarized in a table under methods. | The length of the introduction has been reduced as advised by deleting some material. We have also had to add new material in response to reviewers’ comments. |
| 8. | In p. 6, line 119, the authors refer to “traditional healthcare providers?” I believe the author later refers to these as people who practice holistic medicine (referred to in some places as “traditional healer” or “village doctor”)? If so, the authors should use a consistent label for this group, and possibly place this term in quotes or clearly describe these providers. | The manuscript has been revised to consistently use the term “traditional healers”. We have also defined the type of provider included in this term. (p4, line 91) |
| 9. | p. 10, Time Intervals methods: It is unclear how “estimations” were performed or how much estimation was required (rather than a clear calculation of duration time intervals). Although this may be needed for duration from symptoms to seeking care, this is concerning for recall bias (particularly in other intervals) if based only on patient or family member recollections. | We have replaced the word “estimations” with “calculations”. We have described the timepoints (p. 8, line 173-177) and have commented on potential bias in the Limitations section (p. 17, line 366-368) |
| 10. | Methods: Definitions of how urban and rural are defined are not clearly stated. | Definitions of urban and rural area is added (p. 7, line 143). |
| 11. | Table 5, p-value for marital status should be 0.98 (decimal omitted) | Corrected. |
| 12. | Table 6: ranges in duration are typically provided in addition to medians. This is addressed for some of these in the discussion but should be included in the results section rather than the discussion. | Ranges added in Table 6 as part of the results section. |
| 13. | The authors do not report the stage of lung cancer at the time of diagnosis. Most lung cancers diagnosed in symptomatic patients are at an advanced stage, but it would be of important to know at what stage(s) these patients were diagnosed in order to compare that to other studies, which may vary in stage at diagnosis (which has been associated with variances in time from diagnosis to treatment, for instance, and for which variations in these durations can variably impact outcomes). | We did not ask for this information. Based on the first author’s professional experience, the stage of diagnosis is not usually available in clinical files in Bangladesh. This is generally because of the high cost involved in tests required. Therefore, the information was not requested in the survey. (p. 9, line 210) |
| 14. | Other spelling errors: p. 18, Line 391, should be “finding.” P. 19, line 395 should be “healers.” P. 19, line 402: should read “symptoms.” | Corrected. |
| 15. | Some of the subjective writing in the discussion and conclusion needs explanation. For instance, in the conclusion, the authors note that “informal care was convenient and easy to access, which…” Were these quotes from patient interviews (qualitative research component), or the writers’ beliefs? If so, they should be presented as hypotheses or potential causes rather than facts. For instance, distrust of established healthcare services and fear of diagnosis have been identified as barriers to care/delays in care in some qualitative research studies. | The conclusion is suggesting that the shorter timepoints reflect the ease of accessing informal providers. We have made this clearer.(p. 18, line 381). |
| 16. | Avoiding attribution of cause will help with the authors’ later point that “there is a need to better understand the reasons for the intervals…” | We hope the above point is clearer now and the statement is acceptable. |
| 17. | The figure needs to be more crisp (did not show up well/was blurry on the provided pdf formatting). | Better resolution figure is provided. |
|  | **Reviewer #2** |  |
| 18. | The study methodology was sound enough and the article was well written in palatable sentences that can meet the publication criteria in this well reputed journal, PLOS One. The analysis was good as well. | Thank you. |
|  | **Reviewer #3** |  |
| 19. | I would be interested in knowing the relationship of the authors (all Australian) with the people they worked with in Bangladesh. I found it somewhat surprising that no Bengali collaborator was included on this manuscript and would consider adding one if possible. This does not necessarily need to be included in the manuscript, although I would like a comment in the response. | The first author (AA), who is a Bangladeshi born international student in Australia, is a PhD student supervised by rest of the authors who are Australians. The last author (MAR), one of the supervisors of AA, is a Bangladeshi Australian, who was a Medical Doctor in Bangladesh and familiar with health system there. In addition, the local supervisor is Professor Mohammad Sarwar Alam, who is acknowledged for the support in this project. |
| 20. | For those who are not familiar with the term “informal healthcare providers”, please provide explanation or example in the abstract. | Text added in abstract (p. 2, line 29-30). |
| 21. | The wording in the sentence “Although only one fifth the size of its neighbor 92 Pakistan in geographical area, it has just on 75% the population of that country.” Is odd on the bolded section. Perhaps this should read “geographical area, the population is only slightly smaller (75%) that Pakistan’s population” | Text has been removed as part of the requirement to shorten the introduction. |
| 22. | I really appreciated the detailed context on the Bengali lung cancer statistics and the health system. | Thank you. |
| 23. | I am a little confused about how “traditional healthcare providers” are informal healthcare providers. To me this term indicated traditional hospital-based. I wonder if this is mean to include more of the village doctors? Can you define or clarify this term. I see this is defined later… I might take out the word healthcare or at least move the examples up. Later this is called a traditional healer (which may be a good term) | In some countries (such as China) traditional healthcare providers are hospital based but not in Bangladesh. Traditional healthcare provider/healers and village doctors are different in Bangladesh. Village doctors are unlicensed and unaccredited healthcare providers who practice western medicine in Bangladesh. Traditional healthcare providers include those professionals who practice ayurvedic, homeopathic, unaine/kabiraji (herbal medicine). We have provided more information in the manuscript and have used the term “traditional healers” consistently. |
| 24. | The introduction is very long. It could be shortened by removing some of the non-specific information about lung cancer since there is ample in the discussion of lung cancer in Bangladesh | We have made substantial changes to the Introduction in response to all reviewers’ comments, including shortening it. |
| 25. | It would be helpful to know how often the providers did or didn’t refer. Was this all-consecutive patients? It would be helpful to be able to know a little about potential selection bias. Did all providers refer? | All of our participants had been referred after diagnosis to the tertiary care facility to initiate treatment. Text added (p. 12, line 245-246)  The study sample was consecutive patients who were enrolled if they full-filled the selection criteria and agreed to participate within the time frame of the study. We have added this to the manuscript (p. 5-6, line 116-119) |
| 26. | Was there any patient compensation? | There was not patient compensation, it was voluntary participation. Mentioned in p. 6, line 125. |
| 27. | Please comment on the predominance of men in the study. | Text added (p. 15, line 297) |
| 28. | There is a missing ( in line 279. | Corrected. |
| 29. | This would be a little easier to read with tables at the end of the manuscript. | As per the instruction of the PLOS ONE manuscript format, tables should be included directly after the paragraph in which they are first cited. |
| 30. | The discussion is also quite long. It would be nice to consolidate some of the pharmacy discussion, as this is included with references in separate paragraphs. | Text reduced |
| 31. | In limitations, please commend on how this sample compares to all people getting a diagnosis. I suspect this is a limited sample, which should be included as a limitation. | Text added in the limitation section. |
|  | **Reviewer #4** |  |
| 32. | Why do the authors think the majority of the population is male? Are women not seeking care? Do they not smoke? Are they not seeking care at all? Mentioning this in the discussion section would be helpful. | Text added (p. 15, line 297) |
| 33. | Line 92: sentence is awkward, would revise. “it has just on 75% the population of that country”. Would suggest: “Bangladesh is geographically much smaller but has 75% of the population of Pakistan.” Or something similar | Text has been removed as part of the requirement to shorten the introduction. |
| 34. | Line 191 & 208: typo in “minimise” should be: “minimize” but I defer to the editor | PLOS ONE author guidelines say that British English is preferred. |
| 35. | Line 257: would say “less than or < 45 years” rather than “under 45 years” | Corrected. |
| 36. | Line 257: rural areas. The area needs an (s). | Corrected. |
| 37. | Line 282: end sentence after cancer diagnosis and remove “of lung cancer”. This is redundant. | Corrected. |
| 38. | Table 5: please explain if the p-value is for the distribution of the covariates within the column rather than comparing the urban percentages across the row between formal and information. This can be denoted as a footnote. | Text added as footnote to table to indicate the p-value reflects chi-square tests comparing distribution of each demographic variable across formal and informal healthcare seeking (p. 14, line 272-273). |
| 39. | Line 391: “finding” is misspelled. | Corrected. |
| 40. | Line 406: Recognize rather than “recognise” but I defer to the editor | British English is preferred. |
|  | **Reviewer #5** |  |
| 41. | I note all of the guidelines quoted in the introduction section. It would be good to see any kind of guidance designed for lower income settings, as it is difficult to apply the standards of very wealthy countries to poorer settings (perhaps something from IARC instead?). | We have reduced the amount of text about Guidelines as part of the need to shorten the introduction. Text added (p.3, line 60-64) |
| 42. | I am not sure how relevant some of the other references are either, for example reference 13 on changes in presenting symptoms – these are very UK specific and are not really applicable to LMIC settings. I would be inclined to restructure the introduction de-emphasise the western country examples – I know there is some LMIC material, but I think there is too much from developed countries. | Introduction has been restructured and LMIC reference included |
| 43. | Reference 21 should be updated, there must be some more recent information on the numbers of hospitals providing cancer treatment in the country. | Reference updated (p. 4, line 86) |
| 44. | It would be good to know which literature informed the questionnaire; was it adapted from any existing instrument? Was there any formal validation process? | Reference added for literature and general statement about the type of literature (p 6. Line 127).  As a part of validation process, the questionnaire went through pilot testing (p. 6 line131-133). |
| 45. | It might be good also to describe the provision of primary care in Bangladesh – for example, whilst general practitioners are referred to in the paper, this can mean something quite different in LMIC’s. Typically not many medical doctors are classified as general practitioners. | Brief description added on primary healthcare provision and general practitioners (p 4, line 82 – 86). |
| 46. | Date of first symptom can be very difficult to define with lung cancer. It often occurs on the background of chronic respiratory symptoms and the change can be very subtle eg in the nature of the cough or some additional chest pain. Sometimes there is little change at all in symptoms which may have been present for 20 years or more. This should be highlighted in the discussion section along with comments on the validity of the self-reports used. | Text added about limitations of self-report and complications associated with symptoms (p. 17, line 366 – 368) and (p. 14, line 290-296). |
| 47. | Date of first presentation can also be difficult to define in the context of multiple or long lasting symptoms and there is often a discrepancy between what patients say and primary care records. It may be helpful to refer to some of the methodological diagnostic intervals work – eg the papers on appraisal intervals by Fiona Walter or the Aarhus statement on diagnostic interval studies. The International Cancer Benchmarking Partnership has included lung cancer in its work and that may be worth referring to as well. | Thank you, suggested paper cited (p. 3, line 56-59) |
| 48. | There should be more emphasis on the huge amount of chronic respiratory disease which goes undiagnosed in Bangladesh. While I appreciate the authors acknowledge this limitation, there should be greater recognition that much lung cancer remains undiagnosed, particularly in poor, rural and remote populations in South Asia. | Text added (p. 17, line 353-355). |
| 49. | I note the recommendation for awareness raising campaigns. I am not sure how meaningful such recommendations would be in the context of the study. As partly highlighted in the paper, there are enormous financial, cultural and social barriers to presenting with lung cancer symptoms in LMIC’s. | Text removed. |
| 50. | Some authors describe the interval between first noticing a symptom and reporting it, even reporting it to a family member; diagnostic journeys can be rather convoluted and certainly not linear. There is no mention in the paper of stigma, yet this has been recognised as an important barrier to presenting with symptoms that may indicate cancer in LMIC’s. | We have acknowledged these issues in the Discussion (p. 14, line 290-296). |
| 51. | It would be good to see some recommendations which are a little bit more tailored to the local financially constrained environment. For example, investment in basic primary health care and use of community health workers can help uncover the huge burden of chronic respiratory disease which is often undiagnosed and unmanaged. Any programme targeting lung cancer would need to be undertaken within this broader context of chronic respiratory disease. Treatments for lung cancer are expensive as are diagnostic investigations such as CT scanning and bronchoscopy. It would be better to ground any recommendations for improved diagnosis of lung cancer in the reality of the current capacity in Bangladesh. What kind of investment might achieve the most in settings where treatments such as chemotherapy, radiotherapy and thoracic surgery are severely limited? | Thank you for this suggestion. We have added a recommendation regarding PHC and community health workers. (p. 17, line 356-361).  We have referred to fast-tracking access to formal healthcare as a low-cost recommendation to encourage earlier intervention. |
| 52. | The paper is, nevertheless, of interest because we have very little information to date on cancer diagnostic intervals in South Asian countries and this would be an important addition. I think if it could be better grounded in the international diagnostic interval literature, and if more attention could be paid to local health system and individual factors which might limit any improvements, the paper would be considerably improved. | Thank you for your suggestions. We hope that the extensive changes we have made to the manuscript have covered these points |
|  | **Reviewer #6** |  |
| 53. | Abstract:  As the care seeking trajectory of lung cancer has been studied extensively, please explain why a study Bangladesh is needed. | Text added in abstract. |
| 54. | Please specify “other countries” in the conclusion, are these developing countries or western countries? | Text added (p. 2, line 44) |
| 55. | The introduction is quite lengthy. Some suggestions to shorten it:  Please shorten the description of the recommended timeframes in guidelines. Maybe just describe the range or mean for the different time intervals. | Text has been reduced and restructured in response to all reviewers’ comments |
| 56. | The part about presenting symptoms seems to be a bit out of place and could be left out, as the main presenting symptoms of lung cancer are well-known. Unless you have a hypothesis why presenting symptoms would be different in Bangladesh? Then you should also compare them to the literature. | The discussion around symptoms is revised, we have also had to add new material in response to reviewers’ comments. We hope the discussion is clearer now and acceptable. |
| 57. | I found it very interesting to read about the health system in Bangladesh, it definitely helps to put the results in perspective, so this should remain part of the paper. However, details not directly relevant to the research question, such as e.g. the population of Pakistan, could be left out. Besides, the number of hospitals is based on an article from 2013, maybe more recent data are available? | We have modified the introduction extensively.  Updated reference added. |
| 58. | As to the research questions, I am missing the ‘why’ for the first two. Why is it important to look at the symptoms that motivated patients to seek care and which healthcare providers they visited? What problem could it solve? I would say your main question is the timeline of the care seeking pathway. A subquestion would then be whether some symptoms lead to a longer care seeking pathway as raising awareness about these symptoms could possibly shorten this time. Another subquestion would be for which health care providers the timeline is longer and then find ways to improve this. | Text edited around discussion on how initial symptom and triggering symptoms are different (p. 14, line 283 - 286).  Table 3 has been revised (focusing on whether some symptoms lead to a longer care seeking pathway). |
| 59. | Methods:  Page 8, line 189: A part of questionnaire is about “history of illness”, what exactly does this mean? comorbidities? | Yes, it is comorbidities (text added, p.6 line 138). |
| 60. | - Page 10, line 218: Please further describe how the different time-points were determined. E.g:. -date of first symptoms: was this as reported by the patient in the questionnaire? Based on the medical file? - date of first contact with a health care provider: also including traditional health care providers and pharmacy? - date of diagnosis of lung cancer: either histological or radiological? - date of referral for treatment: by which health care providers and to whom, which treatments included? I assume surgery/radiotherapy/chemotherapy? - date of treatment initiation, which treatments included? | Text added, p. 8, line 173 - 177. |
| 61. | Page 10, line 222: Instead of the long text explaining the different time intervals, I would rather suggest a graph. | Text reduced. |
| 62. | Please use subheadings to structure the results. | Subheading added |
| 63. | Page 11, line 253: Very impressive inclusion rate! Do you know the reason for refusal in the 6 patients? | Potential participants were not required to explain the reason for refusal; however, they were too frail to participate and nominate a caregiver. |
| 64. | Table 1: What is the reason not to test for differences between locations in patient characteristics? | The sample strategy considered variations across study sites but did not seek to establish them. We assumed that hospitals in Bangladesh have different patient profiles (SES, regionality) and selected these three study sites to ensure a good representation of the population. |
| 65. | Table 3: This table could be left out, as it does not add very much to the whole story. Rather, it could be interesting to add whether the timeframes differ by symptoms at diagnosis. | Table 3 is revised to how timeframes differed by symptoms at diagnosis. |
| 66. | Page 14, line 295: It is mentioned that the additional health care professionals visited at pre-diagnosis / pre-treatment were much less often traditional healers/village doctors, but there is also a large drop in pharmacy. This should be mentioned. | Mentioned (p. 12, line 251). |
| 67. | Page 14, line 298: Rather describe something like: The type of health care provider who was the first point of contact did not differ by age, sex, marital status or family structure. However, patients from rural areas, illiterate patients and patients from the lowest income groups were more likely to choose an informal healthcare provider as the first point of contact. | Thank you, text edited (p. 12, line 252 - 255). |
| 68. | Table 6: I assume the numbers in the table are days? Please add the number of patients in the header of the two columns? | Yes, all intervals are in day, mentioned in p. 8, line 179.  Number of patients added in table 6. |
| 69. | A lot of different time intervals are discussed. I would prefer to only discuss in the text the 4 time intervals between the 5 time points and not the other overlapping time intervals. Just mentioning them in the figure or table seems enough to me. | Thank you for the suggestion, we believe that in reducing the number of time points we will miss some important findings. Two durations might have some cumulative effect and change the statistical significance level if we only looked at them combined and did not check the intervals separately. Moreover, we decided to have seven intervals so that we could check how this population differs with other studies, many of which use different intervals. |
| 70. | Discussion:  Were informal care providers also included in the other studies that you cite? This could explain the shorter duration between first onset of symptoms and first contact with a healthcare provider. | There is scarcity of literature where informal healthcare provider is explored as first contact for lung cancer. Made clearer in text (p. 15, line 308). |
| 71. | The comparison with existing literature is quite extensive and could be shortened, while the strengths and weaknesses of the study are hardly discussed and could be given more attention. | Text added in the limitation section. |
