# Supplementary material for: Duration of intervals in the care seeking pathway for lung cancer in Bangladesh: A journey from symptoms triggering consultation to receipt of treatment
Source: PLoS One. 2021 Sep 10;16(9):e0257301. doi: 10.1371/journal.pone.0257301 (PMC8432814; doi:10.1371/journal.pone.0257301)
Supplement: Attachment — Submitted filename: Response to Reviewers.docx [file pone.0257301.s004.docx]

PONE-D-20-39263R1 Response to reviewers’ comments:

NOTE That page and line numbers refer to the clean version of the Revised Manuscript (without tracked changes)

|  | **Feedback** | **Response** |
| --- | --- | --- |
|  | **Journal Requirements:** |  |
| 1. | Please review your reference list to ensure that it is complete and correct. If you have cited papers that have been retracted, please include the rationale for doing so in the manuscript text or remove these references and replace them with relevant current references. Any changes to the reference list should be mentioned in the rebuttal letter that accompanies your revised manuscript. If you need to cite a retracted article, indicate the article’s retracted status in the References list and also include a citation and full reference for the retraction notice. | We have checked the cited papers are available and that none have been retracted. |
|  | **Reviewer #1** |  |
|  | The authors present a study of provider and referral utilization in Bangladesh and the impact on wait times between symptoms to diagnosis and ultimately treatment. The authors' have adequately responded to the reviewers' critiques, leading to improvements in the manuscript clarity and impact. Minor concerns remain as detailed below, one of which includes a concern about the provided Figure 1 | Thank you. |
| 2. | p.13, line 292- In Table 1, it appears the upper quartile had a monthly income of BDT >/= 100,001, but the text states this upper quartile was had monthly income BDT 50,000. Could the authors please correct or clarify? | Thank you, corrected (p.9, line209). |
| 3. | p. 23, line 507: The authors cannot directly suggest that informal care was “convenient and easy to access,” as this study did not evaluate reasons for self-referral to informal care providers. At most, the authors can highlight that most patients’ first point of care is through informal care providers, possibly due to convenience and ease of access (as previously described). The authors can also point out that patients were seen more quickly after initiation of symptoms when the first point was care was an informal care provider. | Thank you, text rephrased (p.18, line 381). |
| 4. | P23, line 511: Do the authors want to highlight that informal care led to significant delays in lung cancer diagnosis and initiation of treatment (not just initiation of treatment alone as it is currently written)? | Thank you, text added (p18, line 384). |
| 5. | Figure 1: Unclear why data for “Onset of symptom to diagnosis” is included twice in this figure. Should the bottom value be “Onset of symptom to Treatment”? | Thank you, corrected. |
|  | **Reviewer #2** |  |
|  | Well written article of a good study. In future other studies may include many more centre to describe more details of it. | Thank you. |
|  | **Reviewer #3** |  |
|  | Thank you for addressing comments, manuscript is improved. I appreciated the improved flow and detail within the manuscript. | Thank you. |
